# Non-replication of the association between 5HTTLPR and response to psychological therapy for child anxiety disorders

**DOI:** 10.1192/bjp.bp.114.154997

**Published:** 2016-02

**Authors:** Kathryn J. Lester, Susanna Roberts, Robert Keers, Jonathan R. I. Coleman, Gerome Breen, Chloe C. Y. Wong, Xiaohui Xu, Kristian Arendt, Judith Blatter-Meunier, Susan Bögels, Peter Cooper, Cathy Creswell, Einar R. Heiervang, Chantal Herren, Sanne M. Hogendoorn, Jennifer L. Hudson, Karen Krause, Heidi J. Lyneham, Anna McKinnon, Talia Morris, Maaike H. Nauta, Ronald M. Rapee, Yasmin Rey, Silvia Schneider, Sophie C. Schneider, Wendy K. Silverman, Patrick Smith, Mikael Thastum, Kerstin Thirlwall, Polly Waite, Gro Janne Wergeland, Thalia C. Eley

**Affiliations:** **Kathryn J. Lester**, DPhil, School of Psychology, University of Sussex, and King's College London, MRC Social, Genetic and Developmental Psychiatry (SGDP) Centre, Institute of Psychiatry, Psychology and Neuroscience, London, UK; **Susanna Roberts**, MSc, King's College London, MRC Social, Genetic and Developmental Psychiatry (SGDP) Centre, Institute of Psychiatry, Psychology and Neuroscience, London, UK; **Robert Keers**, PhD, King's College London, MRC Social, Genetic and Developmental Psychiatry (SGDP) Centre, Institute of Psychiatry, Psychology and Neuroscience, London, UK; **Jonathan R. I. Coleman**, MSc, King's College London, MRC Social, Genetic and Developmental Psychiatry (SGDP) Centre, Institute of Psychiatry, Psychology and Neuroscience, London, UK; **Gerome Breen**, PhD, King's College London, MRC Social, Genetic and Developmental Psychiatry (SGDP) Centre, Institute of Psychiatry, Psychology and Neuroscience, London, UK and National Institute for Health Research Biomedical Research Centre, South London and Maudsley National Health Service Trust, UK; **Chloe C. Y. Wong**, PhD, King's College London, MRC Social, Genetic and Developmental Psychiatry (SGDP) Centre, Institute of Psychiatry, Psychology and Neuroscience, London, UK; **Xiaohui Xu**, MD, King's College London, MRC Social, Genetic and Developmental Psychiatry (SGDP) Centre, Institute of Psychiatry, Psychology and Neuroscience, London, UK; **Kristian Arendt**, MSc, Department of Clinical Psychology and Experimental Psychopathology, University of Groningen, The Netherlands; **Judith Blatter-Meunier**, PhD, Department of Psychology, University of Basel, Basel, Switzerland; **Susan Bögels**, PhD, Research Institute Child Development and Education, University of Amsterdam, The Netherlands; **Peter Cooper**, DPhil, School of Psychology and Clinical Language Sciences, University of Reading, UK and Department of Psychology, Stellenbosch University, South Africa; **Cathy Creswell**, DClinPsy, PhD, School of Psychology and Clinical Language Sciences, University of Reading, UK; **Einar R. Heiervang**, MD PhD, Institute of Clinical Medicine, University of Oslo, Oslo, Norway; **Chantal Herren**, PhD, Department of Forensic Psychiatry, University of Basel Psychiatric Clinics, Basel, Switzerland; **Sanne M. Hogendoorn**, PhD, Department of Child and Adolescent Psychiatry/De Bascule, Academic Medical Centre, Amsterdam, The Netherlands; **Jennifer L. Hudson**, PhD, Department of Psychology, Centre for Emotional Health, Macquarie University, Sydney, Australia; **Karen Krause**, DiplPsych, Department of Psychology, Ruhr-Universität Bochum, Bochum, Germany; **Heidi J. Lyneham**, PhD, Department of Psychology, Centre for Emotional Health, Macquarie University, Sydney, Australia; **Anna McKinnon**, PhD, Brain and Mind Research Institute, University of Sydney, Sydney, Australia, and The MRC Cognition and Brain Sciences Unit, Cambridge, UK; **Talia Morris**, BPsych(Hons), Department of Psychology, Centre for Emotional Health, Macquarie University, Sydney, Australia; **Maaike H. Nauta**, PhD, Department of Clinical Psychology and Experimental Psychopathology, University of Groningen, The Netherlands; **Ronald M. Rapee**, PhD, Department of Psychology, Centre for Emotional Health, Macquarie University, Sydney, Australia; **Yasmin Rey**, PhD, Child Anxiety and Phobia Program, Department of Psychology, Florida International University, Miami, USA; **Silvia Schneider**, PhD, Department of Psychology, Ruhr-Universität Bochum, Bochum, Germany; **Sophie C. Schneider**, BPsych(Hons), Department of Psychology, Centre for Emotional Health, Macquarie University, Sydney, Australia; **Wendy K. Silverman**, PhD, Yale University School of Medicine, Child Study Center, New Haven, Connecticut, USA; **Patrick Smith**, PhD, King's College London, Department of Psychology, Institute of Psychiatry, Psychology and Neuroscience, London, UK; **Mikael Thastum**, PhD, Department of Psychology and Behavioural Sciences, Aarhus University, Aarhus, Denmark; **Kerstin Thirlwall**, DClinPsy, School of Psychology and Clinical Language Sciences, University of Reading, UK; **Polly Waite**, DClinPsy, School of Psychology and Clinical Language Sciences, University of Reading, UK; **Gro Janne Wergeland** MD, Department of Child and Adolescent Psychiatry, Haukeland University Hospital, Bergen, Norway; **Thalia C. Eley**, PhD, King's College London, MRC Social, Genetic and Developmental Psychiatry (SGDP) Centre, Institute of Psychiatry, Psychology and Neuroscience, London, UK

## Abstract

**Background**

We previously reported an association between 5HTTLPR genotype and outcome following cognitive–behavioural therapy (CBT) in child anxiety (Cohort 1). Children homozygous for the low-expression short-allele showed more positive outcomes. Other similar studies have produced mixed results, with most reporting no association between genotype and CBT outcome.

**Aims**

To replicate the association between 5HTTLPR and CBT outcome in child anxiety from the Genes for Treatment study (GxT Cohort 2, n = 829).

**Method**

Logistic and linear mixed effects models were used to examine the relationship between 5HTTLPR and CBT outcomes. Mega-analyses using both cohorts were performed.

**Results**

There was no significant effect of 5HTTLPR on CBT outcomes in Cohort 2. Mega-analyses identified a significant association between 5HTTLPR and remission from all anxiety disorders at follow-up (odds ratio 0.45, *P* = 0.014), but not primary anxiety disorder outcomes.

**Conclusions**

The association between 5HTTLPR genotype and CBT outcome did not replicate. Short-allele homozygotes showed more positive treatment outcomes, but with small, non-significant effects. Future studies would benefit from utilising whole genome approaches and large, homogenous samples.

Anxiety disorders represent a significant global health burden due to their debilitating nature and high lifetime prevalence.^1^ They frequently occur early and show high continuity into adulthood.^2^ Cognitive–behavioural therapy (CBT) is the most established treatment option for childhood anxiety, with around 59% of children remitting following treatment.^3^ The short, low-expression allele of the functional serotonin transporter promoter polymorphism, 5HTTLPR, has been associated with heightened anxiety and related traits^4^ compared with the long allele, although findings are somewhat mixed.^5,6^ Gene–environment interaction studies provide evidence that short allele carriers have the poorest outcomes in high stress environments,^7^ but also show the largest benefit in response to low stress or enriching environments.^8–11^ These findings suggest that 5HTTLPR may represent a ‘differential susceptibility’ marker, which acts ‘for better and for worse’.^12^ Some individuals who are more susceptible to the negative effects of poor environments might also be more sensitive to the positive effects of an enriching experience,^13^ and therefore may respond better to the supportive influence provided by psychological therapies. The field of ‘therapygenetics’ – genetic predictors of outcome following purely psychological therapies – is relatively new.^14^ In a previous study, we found that 5HTTLPR genotype was significantly associated with CBT outcome.^15^ SS homozygotes showed a significantly greater reduction in symptom severity than long allele carriers, and were 20% (18.8%) more likely to be free of their primary (all) anxiety disorder diagnoses by follow-up. A handful of other studies have investigated 5HTTLPR and outcomes following a range of psychological therapies, although results have not been consistent.^15–23^ Two studies also found the SS genotype to be associated with more positive outcomes; following a problem-solving intervention with antidepressants in older adults with post-stroke depression,^16^ and exposure-based therapy in adults with panic disorder with agoraphobia.^17^ However, a third study examining CBT in post-traumatic stress disorder found poorer outcomes in SS carriers.^18^ Additional studies in both adults and children found no effect of 5HTTLPR outcome following psychological interventions. Of note, these studies are all of small sample size, and are generally underpowered to detect what is likely to be a small effect of 5HTTLPR genotype. Furthermore, participants in many of the studies were also on concurrent antidepressant or anxiolytic medications. These factors highlight the need for replication in a large sample to further examine the association between 5HTTLPR genotype and outcome following a purely psychological therapy.

Based on our previous research, the aim of this study was to test whether 5HTTLPR genotype was associated with outcome following CBT in a large replication sample of 829 children with anxiety disorders. This sample size far exceeded that required to detect an effect of similar magnitude to that of our previously reported findings, with greater than 95% power at a significance level of α<0.01. We hypothesised that children with the SS genotype would (a) show greater rates of remission at follow-up and (b) show a greater reduction in symptom severity at follow-up than those with the SL or LL genotype. To further increase power to detect an association between 5HTTLPR genotype and outcome and to estimate an upper bound for the magnitude of the effect, mega-analyses were performed on a combined data-set comprising both our discovery (*n* = 496) and replication samples (*n* = 829). Again, we hypothesised that SS genotype carriers would show a more positive outcome following treatment relative to SL or LL genotype carriers.

## Method

### Study overview

All participants come from the Genes for Treatment (GxT) study, an international multisite collaboration designed to identify clinical, demographic and genetic predictors of outcome following CBT for child anxiety.^24^

### Participants and treatment

Participants aged between 5 and 18 years (mean age: 10 years) met DSM-IV criteria for primary diagnosis of an anxiety disorder. Exclusion criteria included significant physical/intellectual impairment, psychoses and concurrent treatment. All participants completed a full course of CBT either as part of a trial or as treatment as usual at one of 11 sites; Sydney, Australia (*n* = 293); Reading, UK (*n* = 199); Aarhus, Denmark (*n* = 112); Bergen, Norway (*n* = 35); Bochum, Germany (*n* = 50); Florida, USA (*n* = 36); Basel, Switzerland (*n* = 46); Groningen, The Netherlands (*n* = 35); Oxford, UK (*n* = 11); Cambridge, UK (*n* = 9) and Amsterdam, The Netherlands (*n* = 3). All treatments were manualised and treatment protocols across sites were comparable for core elements of CBT, including teaching of coping skills, cognitive restructuring and exposure. Treatment modalities fell into three broad groups – individual CBT (39.8%), group-based CBT (46.1%) and parent-supported guided self-help (14.1%). Further sample characteristics and site-specific trial details can be found in the online data supplement to this article.

### Measures

#### Anxiety disorder diagnoses

Anxiety disorders were assessed at three time points; before and after treatment, and at follow-up (3, 6 or 12 months after conclusion of treatment). Diagnoses were made with the Anxiety Disorders Interview Schedule for DSM-IV (ADIS-IV-C/P)^25^ at all sites except for Bochum and Basel, where the German equivalent (Kinder-DIPs) was used.^26^ Clinical severity ratings (CSRs) were usually based on composite parent and child reports, and were assigned on a scale of 0–8 (see Hudson *et al*^24^ for further details). A diagnosis was made when the child met diagnostic criteria and received a CSR of 4 or more. Primary diagnoses included generalised anxiety disorder (GAD; 34.9%), separation anxiety disorder (SAD; 25.1%), social anxiety disorder (20.8%), specific phobias (11.9%), panic disorder (3.3%), obsessive–compulsive disorder (OCD; 2.1%), post-traumatic stress disorder (PTSD; 1.3%), selective mutism (0.2%, in the cases with primary selective mutism, a diagnosis of severe social phobia was also given; the selective mutism was considered by the clinician to be primary, the most interfering), and anxiety disorders not otherwise specified (ADNOS; 0.5%).

#### Ethnicity

Ethnicity was determined by parent-report regarding the ancestry of the child's grandparents. Those with four grandparents of reported White European ancestry were included in the ‘White European subset’ (*n* = 560, see online data supplement).

### Sample collection and genotyping

DNA samples were collected using either buccal swabs or Oragene saliva kits (DNA Genotek, Ottawa, Canada) and extracted using established procedures.^27,28^ 5HTTLPR was genotyped using polymerase chain reaction amplification and agarose gel electrophoresis according to published protocols.^15^ A selection of samples (*n* = 40) were genotyped in duplicate, with consistent results for each attempt. The genotypic distribution of the sample conformed to Hardy–Weinberg proportions (LL: 25.9%, SL: 51.4%, SS: 22.7%; χ^2^_1_ = 0.69, *P* = 0.444).

### Ethical approval

All trials and collection of samples were approved by site-specific human ethics and biosafety committees. Parents provided informed consent, children assent. The storage and analysis of DNA was approved by the King's College London Psychiatry, Nursing and Midwifery Research Ethics Sub-Committee.

### Sample size and power analyses

Participants used in this paper are referred to as Cohort 2, which consisted of 829 participants with clinical data available both at baseline and at least one outcome time-point (post-treatment, a follow-up time-point or both). However, of these, 792 had outcome data at post-treatment; 606 at follow-up.

Power calculations^29^ suggested a sample size of 285 would be required for 95% power to detect an effect of similar magnitude to our previously reported findings (Cohort 1, odds ratio (OR) = 0.4), at a significance level of α = 0.01 using a recessive allelic model. Our sample of >600 has >90% power to detect a smaller effect size (OR = 0.6) at a significance level of α = 0.01, and 80% power to detect an even smaller effect size of OR = 0.7 at α = 0.05.

### Data analysis

#### Definition of outcome variables

Treatment outcome was defined in a number of ways. Participants were first classed as remitters or non-remitters based on the presence or absence of their primary anxiety disorder diagnosis (primary anxiety remission). Treatment remission was measured both at post-treatment, and at follow-up (collapsed to include all follow-up time-points; 3, 6 or 12 months). Remission was also categorised as the absence of all anxiety disorder diagnoses at post-treatment and at follow-up (all anxiety response). Finally, treatment response was defined as the change in primary anxiety disorder severity from pre- to post-treatment, and from pre-treatment to follow-up.

#### Mixed effects models

To investigate the effect of 5HTTLPR genotype on remission, logistic mixed effects models were used including treatment trial as a higher order random effect. For consistency with the statistical analyses used in our previous study, 5HTTLPR genotype was defined by a recessive model, where SS was coded as 1 and SL/LL were coded as 0. Gender, age and baseline severity were included as covariates in all analyses (age and baseline severity centred at the mean). Analyses were conducted for primary anxiety diagnosis remission and all anxiety disorder diagnoses remission separately at post-treatment and at follow-up. Models focusing on the follow-up time-point included the linear and quadratic effects of time as covariates.

Linear mixed effects models were used to investigate the effect of 5HTTLPR genotype on response (change in primary anxiety disorder severity). Again, trial was included as a higher order random effect, and gender, age and baseline severity were included as covariates. Time was included as a covariate for the model testing change from pre- to post-treatment; and both linear and quadratic effects of time were included as covariates in the model for follow-up.

#### Mega-analyses

Finally, logistic and linear mixed models were run using participants from both Cohort 1^15^ and Cohort 2 combined. A total of 584 samples were genotyped in Cohort 1, from children (aged 6–13) who received CBT for an anxiety disorder. In this sample, 496 had sufficient outcome data to be included in at least one analysis. The sample size of 359 reported in Eley *et al*^8^ refers to the White European participants included in the main analysis of response to therapy at follow-up. Further participants were included in analyses of response to therapy at post-treatment, with a total of 496 included in at least one analysis. Participants included in Cohort 1 were recruited at two sites: Sydney, Australia and Reading, UK. Participants who were genotyped in Cohort 1 but whose clinical data were not available at the time of submission were included in Cohort 2. Combining both cohorts increased the sample size, and so the power to detect an association between 5HTTLPR genotype and outcome. Cohort was included as a covariate in these analyses.

#### Multiple testing corrections

For analyses of remission and response in the mega-analysis, we applied a Bonferroni multiple testing correction to account for the three outcome measures (primary anxiety remission, all anxiety remission and treatment response), giving a corrected significance level of *P* = 0.016. However, the outcome measures used in this study are highly related and nested within each other, and all analyses presented test the same core hypotheses; thus, the correction is likely conservative.

All analyses were performed in STATA version 11.^30^

## Results

### Remission rates

**Fig. 1:**
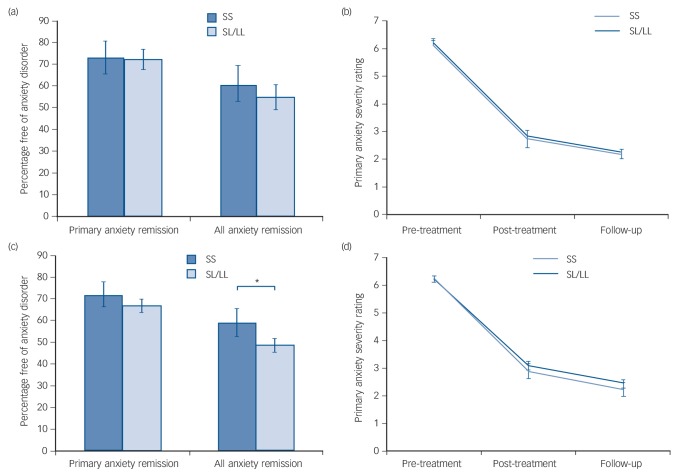
Remission rates and response to cognitive–behavioural therapy (CBT). Figure 1(a) shows the proportion of children free of their primary anxiety disorder and all anxiety diagnoses at follow-up, split by 5HTTLPR genotype in Cohort 2. Figure 1(b) shows the change in primary anxiety symptom severity rating across the course of treatment by 5HTTLPR genotype in Cohort 2. Genotype was not significantly associated with any outcome measure (*P*>0.05 for all analyses). Figure 1(c) shows the proportion of children free of their primary anxiety disorder and all anxiety diagnoses at follow-up, split by 5HTTLPR genotype in the combined sample. Figure 1(d) shows the change in primary anxiety symptom severity rating across the course of treatment by 5HTTLPR genotype in the combined sample. Genotype was not significantly associated with primary anxiety remission or response (*P*>0.05 for all analyses), but those homozygous for the short allele showed a significantly greater remission for all anxiety disorders in the combined sample, even when clinical covariates were taken into account (*OR = 0.45, *P* = 0.014).

Remission rates are given in Table 1 and were marginally higher than in previous studies,^3^ with 61.1% of children remitting from their primary anxiety disorder after treatment, and 71.8% by follow-up. There were no significant differences between remitters and non-remitters for age and gender (age: primary *t*(604) = −0.16, *P* = 0.874; all anxiety *t*(570) = −0.76, *P* = 0.449; gender: primary χ^2^_1_ = 0.42, *P* = 0.515; all anxiety χ^2^_1_ = 0.47, *P* = 0.492). Outcome data by genotype are also given in Table 1 for the additive model, and Fig. 1 shows outcomes for the recessive model. Individuals with the SS genotype were 1.7% more likely than those with SL/LL genotype to remit from their primary anxiety diagnosis and 6.5% more likely to remit from all anxiety diagnoses at follow-up. However, differences between the groups were not statistically significant.

**Table 1:** Treatment outcome (response and remission) in Cohort 2 by 5HTTLPR genotype

Treatment outcome	Time point	Cohort 2	SS genotype	SL genotype	LL genotype
Primary anxiety disorder remission^a^	Post-treatment	61.1	63.7	59.2	62.6
	Follow-up	71.8	73.1	72.3	69.5

All anxiety diagnoses remission^a^	Post-treatment	42.0	40.7	42.0	43.2
	Follow-up	55.7	60.8	55.0	52.4

Primary anxiety disorder response^b^	Post-treatment	3.39 (2.14)	3.47 (2.13)	3.29 (2.14)	3.52 (2.16)
	Follow-up	4.04 (2.21)	4.18 (2.16)	3.98 (2.21)	4.04 (2.24)

Comparisons between genotype groups are not statistically significant for any treatment outcome in Cohort 2.

a. Values for remission are the percentage free of primary and all anxiety disorder diagnoses at post-treatment and at follow-up.

b. Values for primary anxiety disorder response are the mean change in severity from the initial assessment at pre-treatment to the time point specified (s.d. in brackets).

### Logistic mixed effects models – predicting remission

No significant effect of genotype on treatment outcome was detected for either primary or all anxiety diagnoses at post-treatment (Table 2; primary: OR = 0.92, *P* = 0.551; all anxiety: OR = 1.09, *P* = 0.607); or at follow-up (Table 2; primary: OR = 0.83, *P*= 0.318; all anxiety: OR = 0.66, *P* = 0.293). The odds ratio of less than 1 indicates that, as found previously, participants with the SS genotype were more likely to remit than SL/LL children. A similar pattern of effects was seen when analyses were restricted to the White European subset (see Table DS1 in the online data supplement).

**Table 2:** Mixed effect models; categorical remission and change in symptom severity at post-treatment and follow-up for primary anxiety diagnosis; remission from all anxiety disorder diagnoses at post-treatment and follow-up

	Remission						Response – change in CSR		
	Primary anxiety disorder (*n* = 792)			All anxiety diagnoses (*n* = 747)			Primary anxiety disorder (*n* = 788)		
Time point	OR	95% CI	*P*	OR	95% CI	*P*	β	95% CI	*P*
Post-treatment									
Genotype	0.92	0.71 to 1.20	0.551	1.09	0.79 to 1.48	0.607	−0.04	−0.11 to 0.03	0.294
Time							−1.36	−1.41 to 1.30	0.000
Baseline severity	1.15	1.03 to 1.29	0.013	1.22	1.07 to 1.39	0.003	0.29	0.26 to 0.32	0.000
Age	1.02	0.97 to 1.08	0.367	1.00	0.94 to 1.07	0.936	0.01	−0.00 to 0.03	0.083
Gender	1.07	0.86 to 1.33	0.539	1.25	0.97 to 1.62	0.088	0.03	−0.03 to 0.09	0.279

	Primary anxiety disorder (*n* = 606)			All anxiety diagnoses (*n* = 573)			Primary anxiety disorder (*n* = 581)		
Time point	OR	95% CI	*P*	OR	95% CI	*P*	β	95% CI	*P*
Follow-up									
Genotype	0.83	0.35 to 1.99	0.679	0.66	0.31 to 1.43	0.293	−0.04	−0.13 to 0.04	0.318
Time	0.00	0.00 to 0.01	0.000	0.00	0.00 to 0.02	0.000	−1.03	−1.09 to 0.97	0.000
Time^2^	2.72	1.76 to 4.19	0.000	2.27	1.64 to 3.13	0.000	0.15	0.13 to 0.16	0.000
Baseline severity	2.05	1.31 to 3.21	0.002	1.99	1.36 to 2.93	0.000	0.27	0.23 to 0.30	0.000
Age	0.96	0.80 to 1.14	0.631	1.02	0.88 to 1.20	0.750	0.00	−0.02 to 0.02	0.957
Gender	0.79	0.39 to 1.62	0.523	1.20	0.64 to 2.23	0.569	0.03	−0.04 to 0.10	0.461

CSR, clinical severity rating.

5HTTLPR genotype is defined using a recessive model, where SS = 1 and LL/LS = 0. Age and baseline severity are centered at the mean.

### Change in symptom severity

Mean primary anxiety disorder severity at pre-treatment was 6.20 (s.d. = 1.05). At post-treatment, mean severity was 2.82 (s.d. = 2.12), corresponding with a change from pre-treatment of 3.39 (s.d. = 2.14; Table 1). Mean severity at follow-up was 2.15 (s.d. = 2.17) with a mean change from pre-treatment of 4.04 (s.d. = 2.21). There were no significant effects of age or gender on change in symptom severity in Cohort 2 (age: β = −0.01, *P* = 0.55; gender: β = 0.04, *P*= 0.53). Change in severity by genotype is shown in Table 1 and Fig. 1(b). Participants with SS genotype showed a marginally greater reduction in symptom severity by post-treatment (0.10) and by follow-up (0.18) than SL/LL genotype carriers but differences between the groups were not significant.

### Linear mixed effects models – predicting response

No significant effect of genotype on change in primary diagnosis symptom severity was detected, either during the course of treatment (Table 2; pre-treatment to post-treatment: β = −0.04, *P* = 0.294) or at follow-up (pre-treatment to follow-up: β = −0.04, *P* = 0.318). This was also true of the White European subset (online Table DS1). The overall standardised beta coefficient of −0.04 indicates that SS children had a 0.04 s.d. greater reduction in severity scores than SL/LL children.

### Mega-analyses

Before combining Cohorts 1 and 2 for analyses, the two cohorts were compared for key variables. There were no significant differences in gender distribution (χ^2^_1_ = 2.39, *P* = 0.122), treatment modality (χ^2^_2_ = 4.07, *P* = 0.131) or recessive genotype (χ^2^_1_ = 1.98, *P* = 0.159). Due to the recruitment from additional studies in Cohort 2, a larger age range was included and Cohort 2 was significantly older (*t*(1271) = 5.15, *P* = 0.0001). Cohort 2 also showed significantly higher rates of remission at post-treatment (primary: χ^2^_1_ = 7.72, *P* = 0.005; all anxiety: χ^2^_1_ = 7.35, *P* = 0.007) and at follow-up (primary: χ^2^_1_ = 7.24, *P* = 0.007; all anxiety: χ^2^_1_ = 13.60, *P* = 0.000).

In the entire sample (Cohorts 1 and 2 combined), SS participants were 6.6% more likely to remit from their primary anxiety disorder, and 11.4% more likely to remit from all anxiety disorder diagnoses than SL/LL carriers (Fig. 1(c)). Additionally, SS carriers showed a greater reduction in symptom severity from pre-treatment to follow-up than SL/LL carriers (Fig. 1(d); SS: change = 4.01, s.d. = 2.05; SL/LL: change = 3.79, s.d. = 2.19).

When using linear and logistic mixed effect models, the effect of genotype on primary anxiety disorder response and remission did not reach statistical significance (Table 3; change in CSR: β = −0.06, *P* = 0.070; remission: OR = 0.55, *P* = 0.095). However, there was a significant effect of genotype on all anxiety diagnoses remission (Table 3; OR = 0.45, *P* = 0.014), which remained nominally significant when multiple testing corrections were made. In the White European subset, a similar pattern of results was detected, although these did not reach statistical significance (see online Table DS2).

**Table 3:** Mega-analyses; results combining Cohorts 1 and 2. Outcome measures; primary anxiety disorder remission at follow-up, change in primary anxiety CSR from pre-treatment to follow-up, all anxiety disorder diagnoses remission at follow-up

	Remission						Response – change in CSR		
	Primary anxiety disorder (*n* =1044)			All anxiety diagnoses (*n* = 1011)			Primary anxiety disorder (*n* = 1019)		
Predictor variable at follow-up	OR	95% CI	*P*	OR	95% CI	*P*	β	95% CI	*P*
Genotype	0.55	0.27 to 1.11	0.095	0.45	0.25 to 0.88	**0.014**	−0.06	−0.13 to 0.01	0.070
Time	0.00	0.00 to 0.01	0.000	0.00	0.00 to 0.01	0.000	−0.91	−0.95 to 0.86	0.000
Time^2^	2.55	1.94 to 3.36	0.000	2.17	1.73 to 2.72	0.000	0.13	0.11 to 0.14	0.000
Baseline severity	2.05	1.44 to 2.91	0.000	2.30	1.63 to 3.23	0.000	0.27	0.24 to 0.29	0.000
Age	0.95	0.83 to 1.10	0.497	0.99	0.87 to 1.12	0.833	−0.01	−0.02 to 0.01	0.360
Gender	1.21	0.70 to 2.09	0.502	1.33	0.81 to 2.20	0.257	0.05	−0.00 to 0.10	0.070
Cohort	0.65	0.33 to 1.27	0.206	0.55	0.30 to 1.02	0.056	−0.05	−0.12 to 0.01	0.100

CSR, clinical severity rating.

5HTTLPR genotype is defined using a recessive model, where SS = 1 and LL/LS = 0. Age and baseline severity are centred at the mean. Statistically significant results for genotype are highlighted.

## Discussion

We attempted to replicate our previously reported association between 5HTTLPR genotype and outcome following CBT. Our replication sample comprised 829 children with a primary anxiety diagnosis. We found no significant association between genotype and remission of primary or all anxiety diagnoses at post-treatment or follow-up time-points. Furthermore, change in primary anxiety disorder symptom severity (response) across the course of treatment did not differ as a function of 5HTTLPR genotype. Mega-analyses combining data from this and our previous report^15^ revealed a significant association between 5HTTLPR genotype and remission of all anxiety disorders at follow-up. Children carrying the SS genotype were more likely to be free of all anxiety disorder diagnoses than children with the SL/LL genotype. This effect remained significant after multiple testing corrections.

In our previous paper, 20% (18.8%) more children with the SS compared with SL/LL genotypes were free of their primary (all) anxiety disorders at follow-up.^15^ The odds ratios were 0.39 (0.44) for primary (all) anxiety remission. In this study, the effects observed were in the same direction but were markedly smaller and non-significant. This overestimation of effect size within discovery samples is often, at least in part, responsible for the failure of subsequent studies to replicate genetic effects. This is because replication studies are usually underpowered. This study far exceeded the required *N* of 285 needed to detect an effect of OR = 0.39 (as observed in our previous study) with 80% power and α = 0.01. With a sample size of at least 600 in each analysis, this study was sufficiently powered to detect an OR = 0.39 with greater than 99% power and α = 0.001. However, if the true effect of 5HTTLPR in predicting CBT response is closer to the 0.66 observed in the present study, then this would require a sample of *N*>900 to detect this effect at α = 0.01 with 80% power (taking into account the higher remission rate in the Cohort 2).

Mega-analyses are one means of increasing statistical power. To this end, we examined the overall effect of 5HTTLPR genotype on CBT outcome using all data available from this and our previous paper. This allowed us to increase the available sample size to *n* = 1044. The effect of genotype on change in primary anxiety disorder symptom severity was suggestive of an improved response for SS genotype carriers but did not reach conventional levels of significance. We found weak evidence of an association between 5HTTLPR genotype and remission of all anxiety diagnoses at follow-up, which was consistent with our previous findings.^15^ SS genotype carriers were half as likely as SL/LL carriers to retain any anxiety disorder diagnosis at follow-up (OR = 0.45). The odds ratio for primary anxiety disorder remission was in the same direction (OR = 0.55) but did not attain statistical significance. These findings suggest that the 5HTTLPR genotype accounts for only a very small amount of the variance in outcome following CBT. Importantly, the 95% confidence intervals around the OR of 0.45 (for all anxiety disorder remission) suggest that the true effect size lies somewhere between 0.25 and 0.88 and thus effectively provides an upper bound for estimating the effect of 5HTTLPR on remission following CBT. However, given the tendency of early candidate gene discoveries to overestimate the true effect, it remains highly plausible that the true effect may in fact be nearer the lower bound estimate of 0.88 (a higher value represents a smaller effect size with respect to these analyses; see results section for further details). Future research attempting to investigate outcome following psychological treatments as a function of 5HTTLPR genotype should use this information when making decisions regarding appropriate sample size.

The current paper adds to the expanding therapygenetics literature investigating the 5HTTLPR polymorphism.^14^ Although this marker is a plausible candidate for involvement in outcome following psychological therapy, 8 out of 11 studies have failed to find a significant association between the 5HTTLPR and treatment response, with the present study being by far the most highly powered. The remaining studies observed mixed effects: two studies^16,22^ found a significant association between the SS genotype and a more positive treatment response, which is consistent with the mega-analyses reported herein. However, a third small study^18^ reported the opposite with the SS genotype associated with fewer treatment gains. The weak and contradictory findings seen thus far may reflect varying disorder and treatment response phenotypes, the role of medication in some studies and small, underpowered sample sizes.^14^

The use of a semi-structured diagnostic instrument to characterise treatment outcome and agreement across study sites on the definition of response are significant strengths of this study. This study is also the largest to date to investigate genetic predictors of outcome following psychological therapy. However, there are some limitations. First, the sample is very heterogeneous, including a range of anxiety diagnoses and several different CBT modalities. One possibility is that the 5HTTLPR may have greater predictive power when the sample and treatment is more homogeneous.^31^ Second, the current study takes a candidate gene approach. In all areas of psychiatric genetics, candidate gene studies typically report very small effect sizes, often fail to replicate and are sensitive to publication bias.^32^ To be able to nominate candidate genes for investigation also requires a clear understanding of the pathophysiology of the phenotype under investigation and the putative mechanisms through which psychological therapies may act. We know that psychological treatment response is a complex trait, thus it is very unlikely that a single genetic polymorphism such as the 5HTTLPR will explain a sufficiently large amount of variance in outcome to be clinically meaningful in its own right. Although there remains a place for adequately powered studies of plausible candidate genes within the therapygenetics field, this challenge means it will become increasingly important to move towards a whole-genome array-based ‘therapygenomics’ approach. Whole-genome studies are hypothesis free and thus have the potential to identify completely novel and unexpected variants associated with psychological treatment response. Perhaps, most importantly, there is the ability to move beyond single variants and to identify groups of markers and important biological pathways and systems, which collectively capture a (clinically) significant proportion of the variance in outcome. However, it is important to note that some genetic variants (including the 5HTTLPR) cannot be adequately inferred from array data and will continue to need to be genotyped directly. Given the importance of clinical and demographic predictors,^24^ optimal prediction of CBT response is likely to come from analytic approaches, which aggregate genetic information with clinical and demographic variables.^33^

In summary, this study failed to replicate our previously reported association between 5HTTLPR genotype and outcome following CBT for child anxiety disorders. Consistent with our previous findings SS genotype carriers had a more positive treatment response compared with SL/LL genotype carriers. However, these effects were very small and mostly non-significant. The increased sample size afforded by a mega-analytic approach revealed a significant association between SS genotype and remission of all anxiety disorders, which survived multiple testing corrections. Importantly, this work provides an upper and lower bound for estimating the effect size of association between 5HTTLPR and CBT outcomes, which are informative for other researchers working in this area. Going forward, the therapy-genetics field needs to embrace whole genome approaches and to look to recruit large, homogenous samples in which to explore genetic predictors of outcome following psychological therapies.
